# Computed tomography-based automated measurement of abdominal aortic aneurysm using semantic segmentation with active learning

**DOI:** 10.1038/s41598-024-59735-8

**Published:** 2024-04-18

**Authors:** Taehun Kim, Sungchul On, Jun Gyo Gwon, Namkug Kim

**Affiliations:** 1 Department of Convergence Medicine, Asan Medical Institute of Convergence Science and Technology, Asan Medical Center, University of Ulsan College of Medicine, 88, Olympic-ro 43-gil, Songpa-gu, Seoul, 05505 Republic of Korea; 2 Artificial Intelligence and Robotics Institute, Korea Institute of Science and Technology, Seoul, Republic of Korea; 3 Department of Biomedical Engineering, Asan Medical Institute of Convergence Science and Technology, Asan Medical Center, University of Ulsan College of Medicine, Seoul, Republic of Korea; 4 Division of Vascular Surgery, Department of Surgery, Asan Medical Center, University of Ulsan College of Medicine, 88, Olympic-ro 43-gil, Songpa-gu, Seoul, 05505 Republic of Korea; 5 Department of Radiology, Asan Medical Center, University of Ulsan College of Medicine, Seoul, Republic of Korea

**Keywords:** Abdominal aortic aneurysm, Active learning, Application programming interface, Computer-aided design, Deep learning, Endovascular abdominal repair stent graft, Biomedical engineering, Computer science

## Abstract

Accurate measurement of abdominal aortic aneurysm is essential for selecting suitable stent-grafts to avoid complications of endovascular aneurysm repair. However, the conventional image-based measurements are inaccurate and time-consuming. We introduce the automated workflow including semantic segmentation with active learning (AL) and measurement using an application programming interface of computer-aided design. 300 patients underwent CT scans, and semantic segmentation for aorta, thrombus, calcification, and vessels was performed in 60–300 cases with AL across five stages using UNETR, SwinUNETR, and nnU-Net consisted of 2D, 3D U-Net, 2D-3D U-Net ensemble, and cascaded 3D U-Net. 7 clinical landmarks were automatically measured for 96 patients. In AL stage 5, 3D U-Net achieved the highest dice similarity coefficient (DSC) with statistically significant differences (p < 0.01) except from the 2D–3D U-Net ensemble and cascade 3D U-Net. SwinUNETR excelled in 95% Hausdorff distance (HD95) with significant differences (p < 0.01) except from UNETR and 3D U-Net. DSC of aorta and calcification were saturated at stage 1 and 4, whereas thrombus and vessels were continuously improved at stage 5. The segmentation time between the manual and AL-corrected segmentation using the best model (3D U-Net) was reduced to 9.51 ± 1.02, 2.09 ± 1.06, 1.07 ± 1.10, and 1.07 ± 0.97 min for the aorta, thrombus, calcification, and vessels, respectively (p < 0.001). All measurement and tortuosity ratio measured − 1.71 ± 6.53 mm and − 0.15 ± 0.25. We developed an automated workflow with semantic segmentation and measurement, demonstrating its efficiency compared to conventional methods.

## Introduction

Currently, endovascular aneurysm repair (EVAR) is the standard treatment for abdominal aortic aneurysm (AAA)^1^. Post-EVAR complications, including endoleaks, vary depending on the anatomical shape, degree of calcification, and extent of the thrombus in the aorta^2^. Particularly, when EVAR of AAA deviates from the instructions for use (IFU), clinical outcomes, including re-interventions and mortality, are worse than the cases with the IFU^3^. Currently, medical imaging examinations, such as computed tomography (CT), are crucial and the only procedures to evaluate the shape and size of an AAA before EVAR. However, the inter-observer reproducibility of aortic shape measurement is poor at 87%, exceeding the clinical tolerance of ± 5 mm for aortic diameter measurement^4^. This limitation negatively impacts post-procedural clinical outcomes, making automated segmentation and measurement valuable for planning EVAR and improving its overall success.

Recently, deep learning (DL) models, particularly the U-Net architecture, have made major advancements in medical image segmentation. They have achieved significant improvements in accuracy and robustness when segmenting anatomical structures and diseases^5^. These DL advancements have enabled more accurate segmentation of various medical conditions and internal structures of the human body^6–9^. Generally, DL requires many medical images with labeling; however, labeling large amounts of data or complex anatomies is difficult. To address this problem, various studies have introduced active learning (AL) frameworks, which reduce the need for manual annotation. AL divides a dataset into smaller subsets and initiates training. It involves an iterative “human in the loop” process in which a model infers on unlabeled data, and human experts modify the annotations. This continues until the model achieves satisfactory performance or annotation resources^10,11^.

Efficient measurement based on segmentation offers benefits, such as reduced lead times, accurate landmark measurement, and robustness. Automated 3D measurement can be performed by algorithmizing the measurement process based on python-script using tools that are basically embbeded in computer-aided design (CAD) software such as Solidwork, 3-matic, and Rahno^12–14^. The conventional repetitive manual segmentation and measurement are tedious, labor intensive, and time consuming. Furthermore, manual tasks are associated with variations in inter- and intra-human variabilities. Caradu et al.^15^ used an automatic segmentation software to robustly segment the lumen and thrombus in AAA. The segmentation was subsequently manually corrected by senior and junior surgeons. Wyss et al.^2^ generated a central luminal line after the segmentation of CT images and measured specific landmarks on a cross-sectional plane in 2D images for predicting complications. However, they focused on automated segmentation-based detection with two classes and measurement with a 2D image-based cross-sectional plane, potentially causing discrepancies with the actual 3D anatomy.

Unlike previous studies, we developed a semantic segmentation algorithm with AL for AAA using abdominal CT and automated measurement based on the 3D model obtained by the developed semantic segmentation using CAD.

In contrast to prior investigations, our study presents a novel approach by devising a semantic segmentation algorithm with AL for AAA utilizing abdominal CT scans. Moreover, we introduce an automated measurement framework leveraging the 3D model generated through the semantic segmentation process using scripts-based application programming interface (APIs) of CAD. This holistic methodology not only enhances the accuracy of AAA segmentation but also streamlines the measurement process, signifying a substantial advancement in medical imaging analysis for AAA diagnosis and treatment planning.

## Methods

### Dataset acquisition

The retrospective study carried out in accordance with the principles of Declaration of Helsinki and current scientific guidelines. The institutional review board for human investigations at Asan Medical Center approved this study with a waiver of informed consent from patients because of the use of retrospective clinical and imaging data. The data were de-identified, in accordance with the Health Insurance Portability and Accountability Act privacy rule. All methods were performed in accordance with the relevant guidelines and regulations. The dataset could be available on request from the corresponding authors with allowance of our IRB.

Three hundred subjects diagnosed with AAA were enrolled in the Asan Medical Center (AMC) between March 2007 and December 2016. All participants underwent pre-operative CT angiography scanning with a slice thickness of 2.5–5.0 mm, field of view (FOV) of 512 × 512 × z-axis, and pixel size of 0.5781–0.9258 mm (Table 1). From the 300 participants, for 96, automated measurement using CT angiography scans was performed with a tube voltage of 120 kVp, pixel size of 0.5781–0.8164 mm, and slice thickness of 2.5–5.0 mm (Table 1).

**Table 1:** Details of CT scans of enrolled patients.

Dataset	Semantic segmentation	Automated measurement
Subject (N)	300	94
Field of view (mm)	512 × 512	512 × 512
Tube voltage (kV)	80–130	100–130
Pixel size (mm)	0.5781–0.9258 × 0.5781–0.9258	0.5781–0.8164 × 0.5781–0.8164
Slice thickness (mm)	2.5–5.0	2.5–5.0

### Procedure

**Figure 1:**
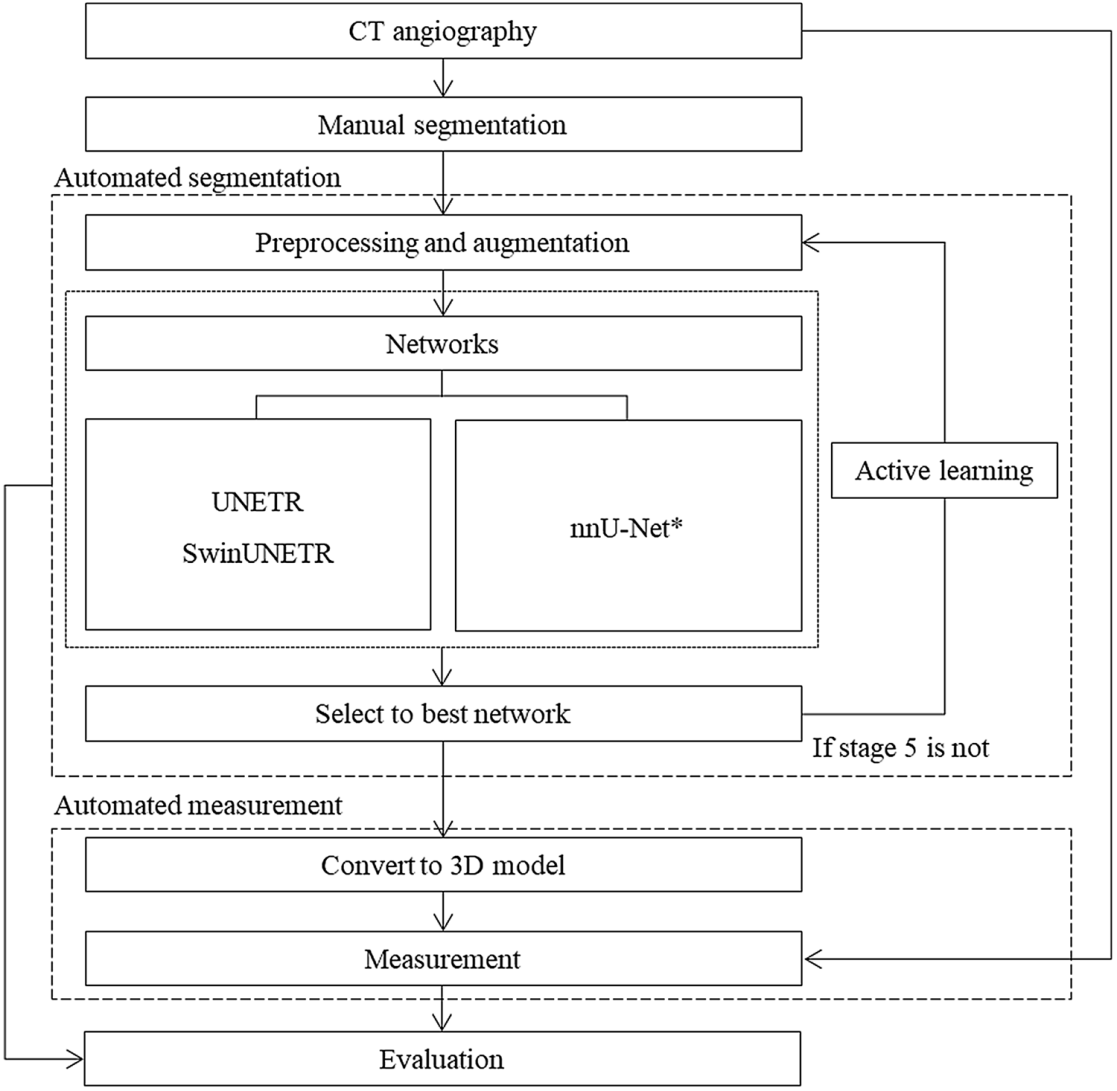
Overall process of semantic segmentation with active learning (AL) and measurement using computer-aided design (CAD). In automated segmentation, after pre-processing and augmentation of CT images, training is conducted using several networks. The best-performing network is selected, and AL is performed up to Stage 5. In automated measurement, based on the data obtained from automated segmentation, the 3D model is generated. Automated measurements are then carried out using a script-based application programming interface (API), followed by evaluation against conventional CT image-based measurements. *nnU-Net is consisted of 2D, 3D U-Net, 2D-3D U-Net ensemble, and cascaded 3D U-Net. (*UNETR* UNEt Transformers, *SwinUNETR* shifted-windows UNEt TRansformers).

The retrospective study was divided into two parts: (1) semantic segmentation and (2) automated measurement (Fig. 1). Initially, abdominal CT images were manually segmented into the aorta, thrombus, calcification, and vessels. These sub-datasets were preprocessed and augmented before training various models: UNEt TRansformers (UNETR)^16^, shifted-windows UNEt TRansformers (SwinUNETR)^17^, and no-new-U-Net (nnU-Net), including 2D U-Net, 3D U-Net, 2D–3D U-Net ensemble, and cascade 3D U-Net^18^. The preprocessing, augmentation, and training processes were repeated on both the original and additional datasets until no new datasets were available for AL with five stages. In the stages, a new dataset was predicted from the previous model and then corrected by human experts in a “human in the loop” process. Three-dimensional models were generated by semantic segmentation, and automated measurement with CAD was conducted with clinically defined landmarks. Finally, performances of the various networks and stages were evaluated and compared to those of the manual measurements by medical doctors.

### Dataset distribution and AL

**Figure 2:**
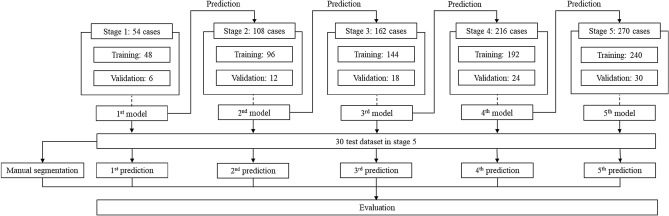
Training and test strategy with active learning (AL). *All stages except Stage 1 contain the same dataset from the training and validation of the previous stage.

All participants were divided into five stages of AL, and the data distribution for the training, validation, and testing is depicted in Fig. 2. In the first stage, the ground truths were manually delineated for 48 training, 6 validation, and 6 test samples from the CT angiography scans of 60 participants, covering four classes. The next stage involved manually correcting the predicted segmentation obtained using a convolutional neural network, resulting in AL-corrected segmentation for 60 new data. A total of 120 subjects were trained (60 from the previous stage and 60 new subjects). Stages 3 and 4 followed a similar process to stage 2, and in the final stage, all 300 subjects were used: 240 as training, 30 as validation, and 30 as test samples. The best-performing network in the final stage was selected, and the best network trained in each stage was used to infer on the 30 test sets. The results were evaluated by manual segmentation (Fig. 2).

### Initial manual and AL-corrected segmentation

In the initial segmentation, the aorta was segmented by “Thresholding” positioning the FOV and “Region growing” with a seed point and “Close” under “Morphology Operations” for filling the outside of the Hounsfield units (HUs) of the aorta. The thrombus with a small amount of an injected contrast medium, small vessels, and calcification were segmented using the “Edit Mask” function to add or eliminate areas. The AL-corrected segmentation used the same techniques as the initial segmentation to correct the predicted binary masks. These masks were then superimposed onto the CT images and manually adjusted to produce the ground-truth segmentation. We used Mimics software (Materialise., Leuven, Belgium) for segmentation of all dataset.

### Preprocessing

The data preparation involved preprocessing steps, including foreground elimination to remove irrelevant regions such as the background. Z-score normalization was performed to adjust the contrast in the CT images, and the intensity values were clipped between 0 and 1^19^. The spatial properties of the images were standardized by resizing the FOV to 512 × 512 × z-axis mm and applying a spacing of 1.0 × 1.0 × 3.0 mm.

### Networks and experimental settings

nnU-Net is a framework for medical image segmentation. It utilizes a self-adapting approach to optimize hyperparameters, including preprocessing, loss optimization during training, and post-processing operations. Nested cross-validation loops are used to enhance the performance of specific segmentation tasks on different data subsets^18^. UNETR and SwinUNETR are medical image segmentation architectures provided by the Medical Open Network for Artificial Intelligence (MONAI) that use transformers^16,17^. UNETR combines U-Net with transformers and employs self-attention. SwinUNETR is an upgraded version optimized for medical image segmentation^17^. It has a hybrid design with a Swin transformer encoder for high-level features and a U-Net decoder for segmentation maps. The Swin transformer breaks down an input image into smaller patches and applies self-attention layers to capture features at various scales. The hyper-parameter of nnU-Net is applied with self-configuration. In UNETR and SwinUNETR, for augmentation, various transformations were performed: foreground extraction, random rotation (90°), random flips (x-, y-, and z-axes), and random intensity shifts with 3D patch (96 × 96 × 96). The training used the Adam optimizer (learning rate = 0.0001, weight decay = 0.00001) with the dice cross entropy loss and a batch size of 4. The training epochs for nnU-Net were set to 10,000, while UNETR and SwinUNETR were set to 100,000 steps for training. The training was performed on an NVIDIA TITAN RTX GPU with 24,220 MiB, using MONAI 0.1.0 and PyTorch 1.12.1.

### Landmarks of AAA

**Figure 3:**
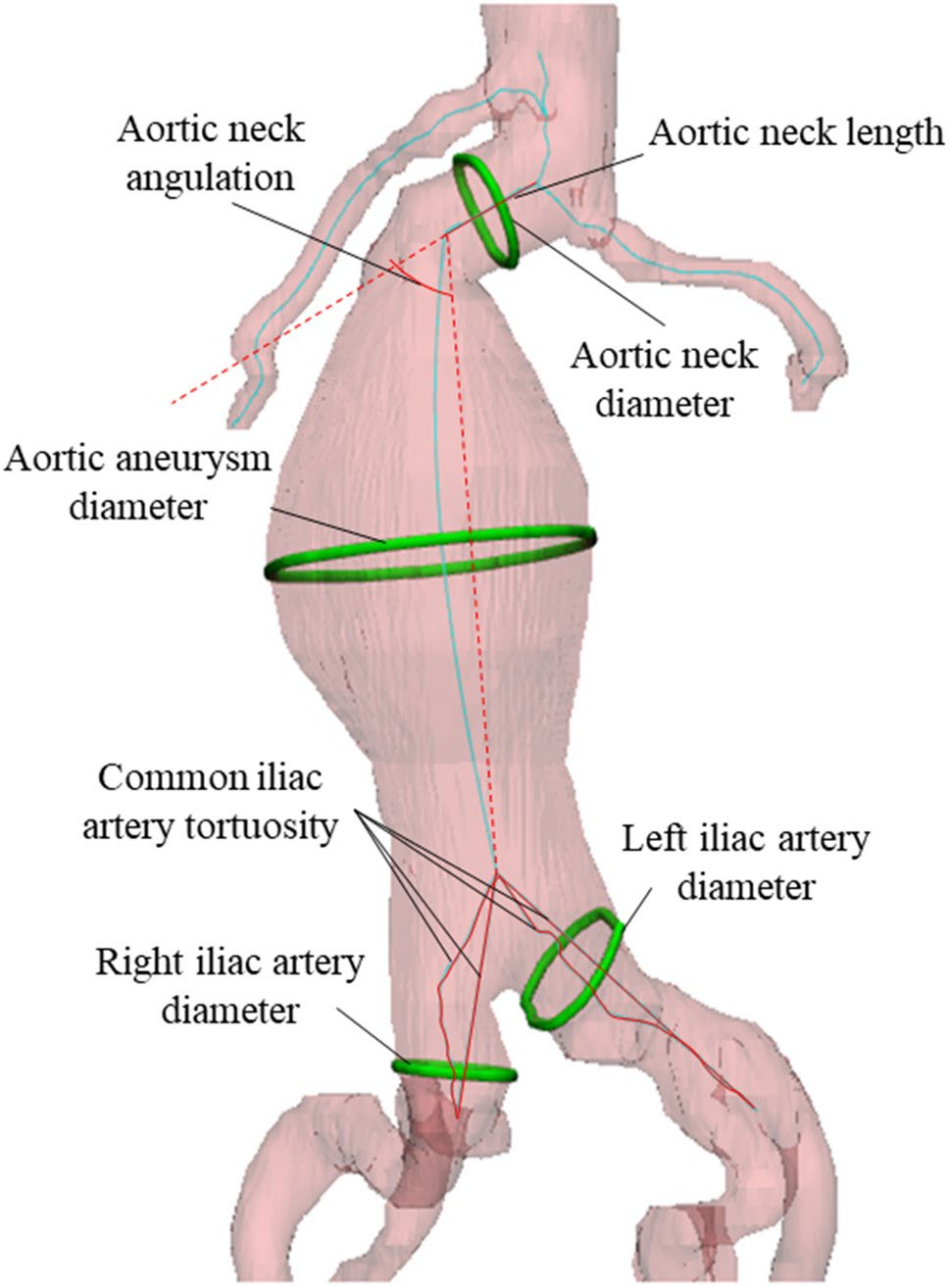
The measurement landmarks for determining the stent graft according to the patient’s anatomy. It was defined by seven landmarks: aortic neck diameter, which is the diameter of the midpoint between the lower part of the renal artery and the starting point of the aneurysm, aortic aneurysm diameter, which is the maximum diameter of the aneurysm area, right and left iliac artery diameter, which is the maximum diameter of the iliac artery, aortic neck length, which is the distance between the lower part of the renal artery and the starting point of the aneurysm, common iliac artery tortuosity, which is the ratio of the centerline and the straight line between the start and end of the common iliac artery, and aortic neck angulation, which is the angle between the aortic neck and aortic aneurysm.

To avoid complications such as endoleaks and re-intervention after EVAR and determine suitable commercially available endografts based on the anatomical size of a patient, a 3D model was generated by automatic segmentation. It was defined by seven landmarks: (1) aortic neck diameter, which is the diameter of the midpoint between the lower part of the renal artery and the starting point of the aneurysm, (2) aortic aneurysm diameter, which is the maximum diameter of the aneurysm area, (3) right iliac artery diameter, which is the maximum diameter of the right iliac artery, (4) left iliac artery diameter, which is the maximum diameter of the left iliac artery, (5) aortic neck length, which is the distance between the lower part of the renal artery and the starting point of the aneurysm, (6) common iliac artery tortuosity, which is the ratio of the centerline and the straight line between the start and end of the common iliac artery, and (7) aortic neck angulation, which is the angle between the aortic neck and aortic aneurysm (Fig. 3).

### Conventional and automated measurement

The conventional measurement was performed with tools of a picture archiving and communication system (PACS) based on 2D images. Seven measurements were performed using the PACS. On the selected axial slice, the measurement included the antero-posterior, transverse, and maximum diameters in any direction. On the selected sagittal slice, the measurement included the antero-posterior diameter and the diameter perpendicular to the long axis of the aneurysm. On the coronal slice images, the measurement included the transverse diameter and the diameter perpendicular to the long axis of the aneurysm^4^. Two algorithms were developed for the automated measurement of the landmarks in AAA, requiring inputs such as a 3D aortic model with a thrombus, a centerline dividing the aortic neck and the aneurysm, right and left iliac arteries, and start planes of the right and left iliac arteries. The necessity of each input is as follows: (1) 3D aortic model serves as the base for all measurements, enabling the generation of centerlines and measurement. (2) Centerlines are utilized in conjunction with the 3D aortic model to measure diameter, length, tortuosity, and angulation. (3) The planes of the right and left iliac arteries are employed to separate the iliac artery model from the aortic 3D model, and the separated 3D models and their centerlines are used to determine maximum diameter.

Algorithm 1 involved generating points along the centerline and the directions between each point and its consecutive one. This process created planes, which were used to intersect the 3D model of the AAA. The longest contour among the generated contours was selected for the measurement. The aortic neck diameter was measured with the midpoint and its consecutive one between the starting and ending points of the aortic neck centerline and lines based on these two generated points. Subsequently, the mid-plane was generated using the midpoint and the direction of the line and then intersected with the AAA 3D model. The maximum diameter of the mid-contour was measured. The aortic aneurysm diameter was obtained by feeding the AAA 3D model and the centerline of the aortic aneurysm into Algorithm 1. Determining the diameters of both iliac arteries necessitates obtaining the starting plane of the centerline for each iliac artery. This involves cutting the AAA 3D model based on the plane corresponding to the right or left side and measuring using Algorithm 1. The aortic neck length was derived by selecting the aortic neck centerline. The tortuosity of the common iliac artery was determined based on the ratio of the straight length and its centerline of the AAA 3D model. The straight length was that between the starting and ending points of the centerline for each iliac artery. Finally, obtaining the aortic neck angulation involved calculating the angle formed by two lines that were derived from the starting and ending points of the centerlines of the aortic neck and the aortic aneurysm (Algorithm 2). The inputs for the automated measurement were as follows: (1) an aortic model with a thrombus, (2) the aortic neck centerline, (3) the aortic aneurysm centerline, (4) the centerline of the right and left iliac arteries, and (5) the planes of the right and left iliac arteries. Supplementary Video 1 was shown in the automated measurement process for 7 landmarks.

**Algorithm 1:**
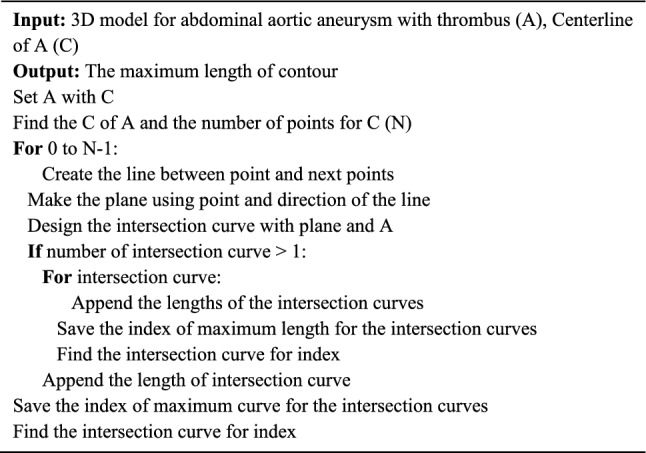
Pseudocode for determining maximum contour.

**Algorithm 2:**
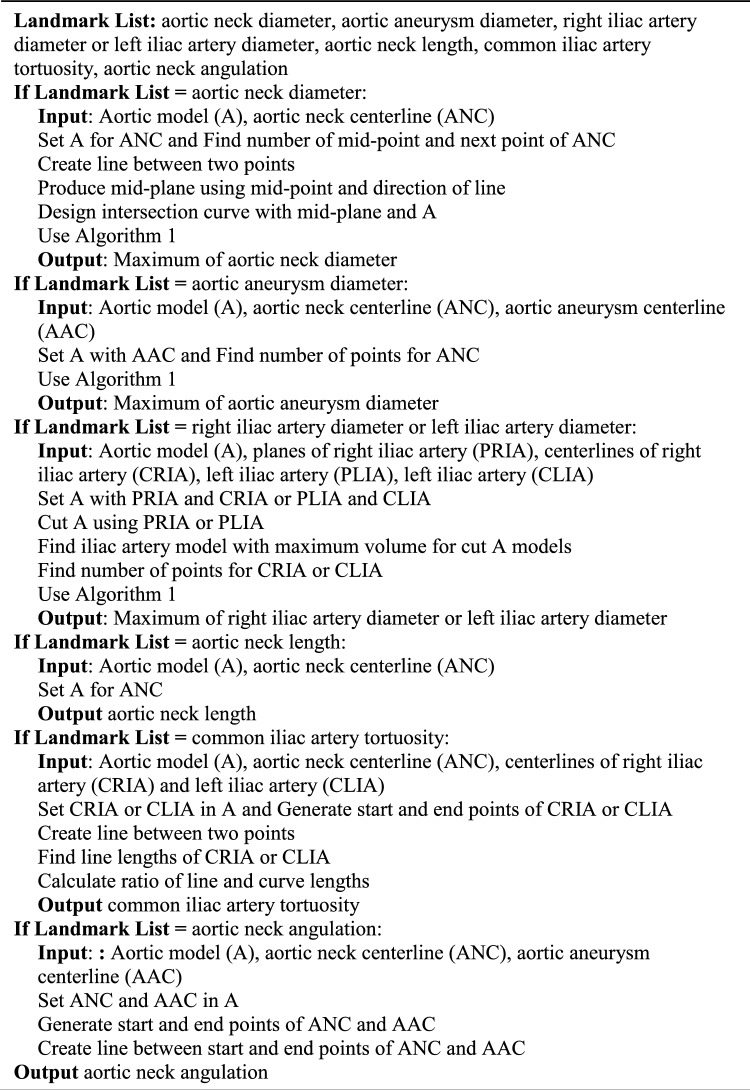
Pseudocode for automatic measurement of seven landmarks.

### Evaluation

To assess the accuracy of the predicted labels against the ground truths in the semantic segmentation, the dice similarity coefficient (DSC) and 95% Hausdorff distance (HD95) were utilized^20^. The DSC is a metric that ranges from 0 to 1, with 0 and 1 indicating no and perfect overlaps between the volumes, respectively. HD95 is similar to the maximum Hausdorff distance and it removes outliers by considering the 95th percentile of the distances. This prevents extreme values from significantly influencing the metric. The saturation concerning dataset sizes was evaluated across various classes, revealing discrepancies in predictive performance. While classes such as aorta demonstrated effective prediction with relatively small dataset sizes, others like thrombus, calcification, and vessels struggled despite larger dataset sizes. By comparing results at each stage, optimal dataset sizes were determined for individual classes, facilitating their respective optimization. In addition, manual and AL-corrected segmentation times by one observer were evaluated on the same ten patients randomly selected from the test set. All statistics were evaluated using paired t-tests. The measurements were evaluated by the Bland–Altman analysis between our method and the manual ground truths obtained by medical doctors with limit of agreement, which used to remove outliers lying outside the 95% range.

In addition, for the centerline, which was divided manually for the automated measurement, human variation was confirmed using three observers and by correlation analysis.

## Results

### DSC and HD95 in semantic segmentation with various networks

**Figure 4:**
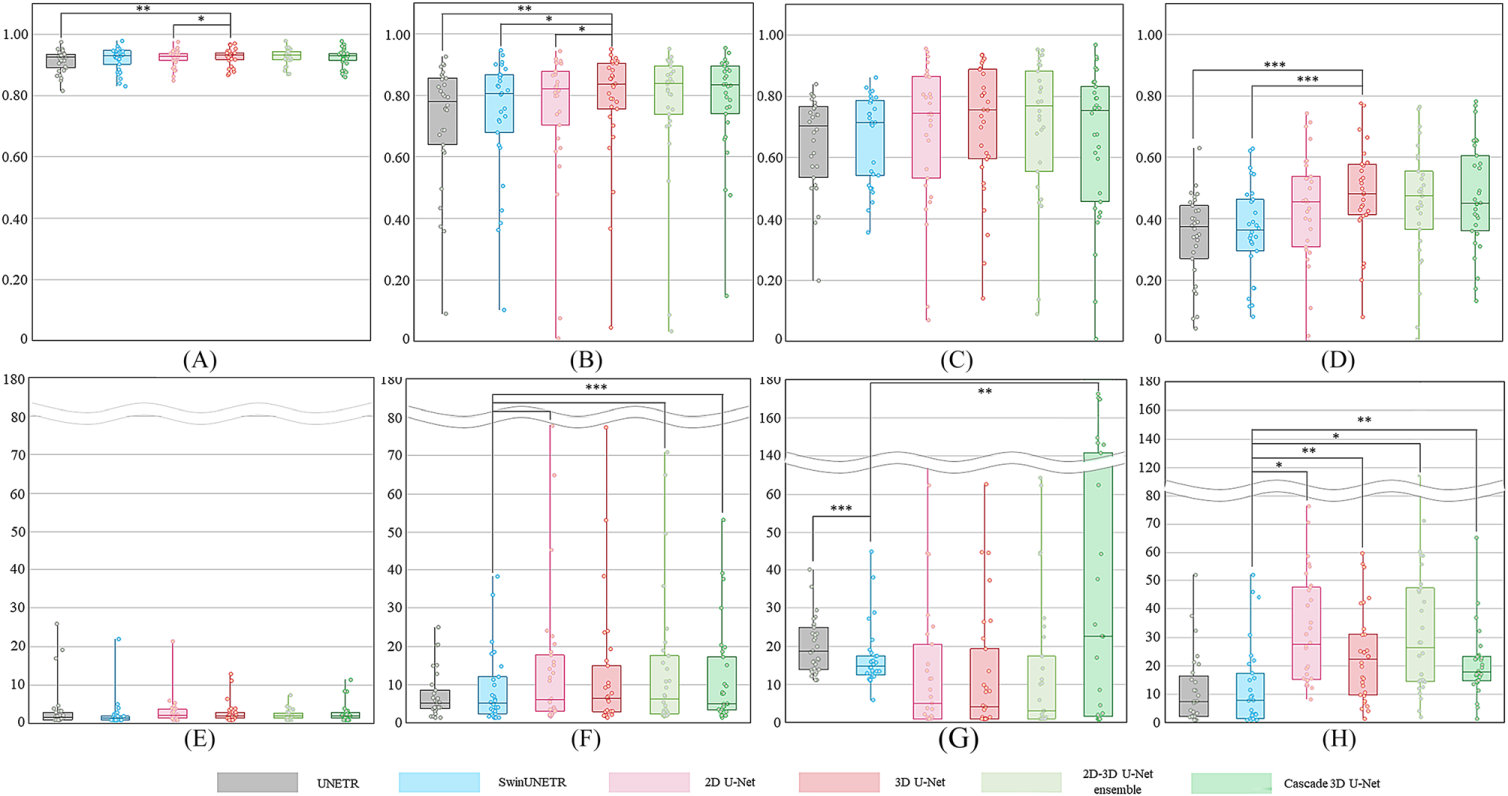
DSC and HD95 of each class in stage 5 obtained using various networks. DSC and HD95 of (**A,E**) aorta, (**B,F**) thrombus, (**C,G**) calcification, and (**D,H**) vessels. Paired t-tests between stage 5 and other stages; *p < 0.05, **p < 0.005, ***p < 0.0005; *DSC* dice similarity coefficient, *HD95* 95% Hausdorff distance, *SwinUNETR* shifted-windows UNEt transformers.

**Figure 5:**
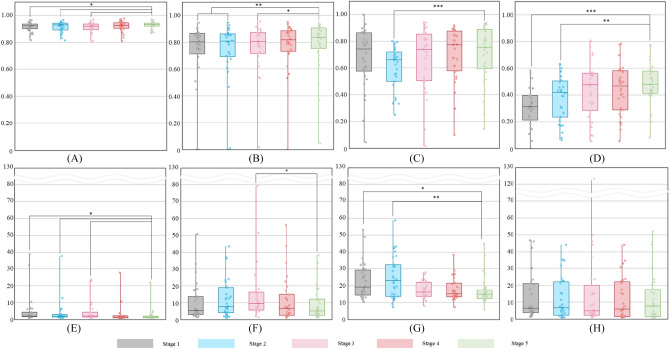
DSC of 3D U-Net and HD95 of SwinUNETR showed the best performance for each class from stage 1 to stage 5. DSC of 3D U-Net and HD95 of SwinUNETR for (**A,E**) aorta, (**B,F**) thrombus, (**C,G**) calcification, and (**D,H**) vessels. Paired t-tests between stage 5 and other stages; *p < 0.05, **p < 0.005, ***p < 0.0005, *DSC* dice similarity coefficient, *HD95* 95% Hausdorff distance, *SwinUNETR* shifted-windows UNEt transformers.

Figure 4 was shown in the boxplot for the DSC and HD95 in stage 5, comparing the different networks. In stage 5, the 2D–3D U-Net ensemble yielded the most accurate outcomes for the aorta and calcification, scoring 0.928 ± 0.026 and 0.702 ± 0.226, respectively. Cascade 3D U-Net achieved the highest accuracy for the thrombus, scoring 0.782 ± 0.170. For vessels, the 3D U-Net was the best with a score of 0.481 ± 0.155. The 3D U-Net showed the highest performance with an average DSC of 0.722 ± 0.227 and exhibited statistically significant differences with all networks (p < 0.01) except for the 2D–3D U-Net ensemble (p = 0.153) and cascade 3D U-Net (p = 0.102). In terms of HD95, for the aorta, the 2D–3D U-Net ensemble achieved the best value of 2.31 ± 1.42 mm, whereas for calcification, the highest accuracy was achieved with in 3D U-Net with a score of 12.39 ± 16.62 mm. UNETR was the best-performance for the thrombus and vessels with HD95 of 7.46 ± 6.12 and 11.61 ± 12.58 mm in stage 5, respectively. SwinUNETR yielded the best outcome in terms of the average HD95, with a value of 10.23 ± 6.19 mm. Statistical analysis indicated a significant difference between SwinUNETR and all networks (p < 0.01) except for UNETR (p = 0.540) and 3D U-Net (p = 0.118) (Fig. 4). The DSC and HD95 values in each stage of 3D U-Net are shown in Fig. 5. Comprehensive results for all networks, including their stages and classes, are summarized in Supplementary Table 1 and Fig. 4.

### Saturation evaluation of 3D U-Net

In each stage of 3D U-Net, the aorta consistently showed a small DSC variation, ranging from 0.909 to 0.926, suggesting performance saturation with only 60 cases in stage 1. By contrast, the thrombus and vessels displayed larger DSC variations, ranging from 0.733 to 0.779 and 0.310 to 0.481, respectively, across the different stages. The performance continued to improve even in stage 5 with a larger dataset of 300 cases. For calcification, DSC values ranging from 0.602 to 0.703 were obtained, showing improvement until stage 4 with 240 cases (Fig. 5, Supplementary Table 1).

### Segmentation times of manual and AL-corrected segmentation using 3D U-Net

**Figure 6:**
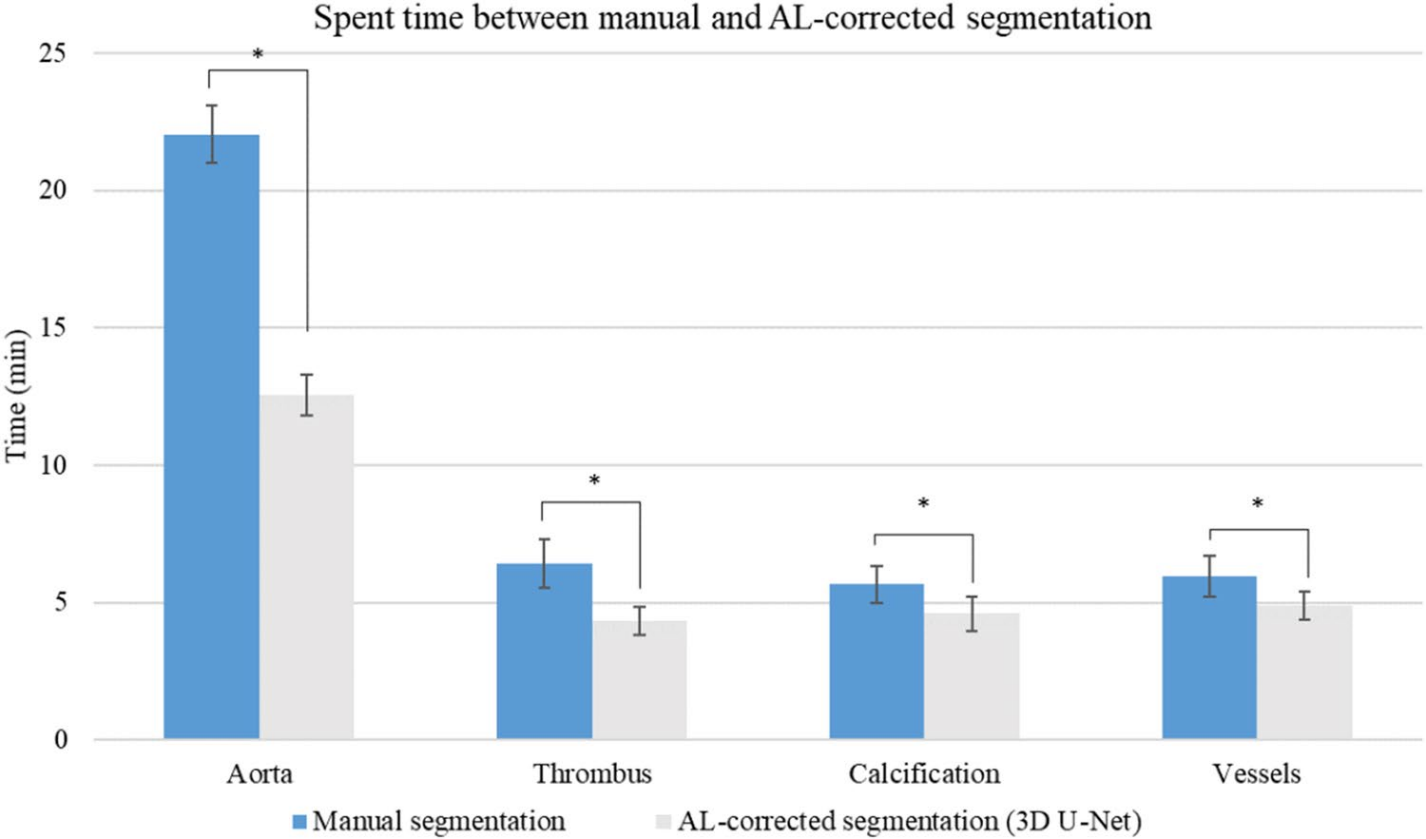
Manual and AL-corrected (using 3D U-Net) segmentation times (*p < 0.001).

The spent time difference between the manual and AL-corrected segmentation using the best model (3D U-Net) reduced as 9.51 ± 1.02, 2.09 ± 1.06, 1.07 ± 1.10, and 1.07 ± 0.97 min for the aorta, thrombus, calcification, and vessels, respectively. These times were statistically significant difference (p < 0.001) (Fig. 6, Supplementary Table 2).

### Comparison of conventional and automated measurements

Table 2 lists the arithmetic means and standard deviations of the differences between the conventional and automated measurements of the aortic neck diameter, aortic aneurysm, right and left iliac artery diameters, aortic neck length, and tortuosity of both iliac arteries (curve length, line length, and ratio). For the angulation between the centerline of the aortic neck and the aneurysm, errors were observed for three patients both in manual and automated measurements, particularly when the angle was not 60°.

**Table 2:** Manual and automated measurements of aortic neck diameter, aortic aneurysm diameter, right and left iliac artery diameters, aortic neck length, and tortuosity.

Landmark	Conventional measurement	Automated measurement	Difference (LoA)
Aortic neck diameter (mm)	23.41 ± 3.25	24.60 ± 4.21	− 1.19 ± 3.92 (− 8.88 to 6.50)
Aortic aneurysm diameter (mm)	55.99 ± 10.02	55.76 ± 10.10	0.23 ± 4.00 (− 7.61 to 8.06)
Right iliac artery diameter (mm)	18.76 ± 6.10	20.90 ± 5.57	− 2.14 ± 4.37 (− 10.70 to 6.43)
Left iliac artery diameter (mm)	16.96 ± 5.61	19.76 ± 5.34	− 2.80 ± 4.09 (− 10.82 to 5.22)
Aortic neck length (mm)	33.06 ± 13.50	35.71 ± 15.98	− 2.65 ± 11.88 (− 25.94 to 20.64)
Tortuosity of right iliac artery			
Curve length (mm)	46.99 ± 12.40	56.93 ± 17.76	− 9.95 ± 12.81 (− 35.05 to 15.16)
Line length (mm)	42.41 ± 11.62	49.49 ± 13.98	− 7.08 ± 10.57 (− 27.79 to 13.63)
Ratio	1.13 ± 0.24	1.14 ± 0.10	− 0.01 ± 0.20 (− 0.41 to 0.38)
Tortuosity of left iliac artery			
Curve length (mm)	52.27 ± 15.13	61.95 ± 17.64	− 9.68 ± 10.96 (− 31.16 to 11.81)
Line length (mm)	61.01 ± 17.64	53.65 ± 13.88	8.30 ± 7.78 (− 6.94 to 23.54)
Ratio	0.86 ± 0.17	1.15 ± 0.15	− 0.29 ± 0.22 (− 0.29 to 0.14)
Angulation			
< 60° (n)	86	89	–
> 60° (n)	10	7	–

*LoA* limit of agreement, *n* number of patients.

### Human variation in automated measurement

For the automated measurement, manual input data was essential. These included a 3D aortic model with a thrombus, the centerline of the aortic model divided by the aortic neck, the aortic aneurysm, right, and left iliac arteries, and the starting planes of the right and left iliac arteries. Human variation in the automated measurement could occur owing to the manual centerline division. The correlation analysis showed a high level of agreement, with an intraclass correlation coefficient value of 1.00 for all measurements, indicating no significant human error in the automated measurement. In addition, all correlations were significant at 0.01 (Table 3).

**Table 3:** Correlation analyses of three researchers for aortic neck diameter, aortic neck length, and right and left tortuosity including curve length, line length, and ratio.

Correlation	r_12_	r_13_	R_23_
Aortic neck diameter	0.999	1.000	0.998
Aortic neck length	1.000	1.000	1.000
Right tortuosity			
Curve length	1.000	1.000	1.000
Line length	1.000	1.000	1.000
Ratio	0.999	0.999	1.000
Left tortuosity			
Curve length	1.000	1.000	1.000
Line length	1.000	1.000	1.000
Ratio	1.000	1.000	1.000

*r*_*12*_ correlation between researchers 1 and 2, *r*_*13*_ correlation between researchers 1 and 3, *r*_*23*_ correlation between researchers 2 and 3; all correlations, p < 0.01.

## Discussion

We developed a semantic segmentation and measurement method for AAA using abdominal CT. The semantic segmentation involves using several DL architectures with AL. The automated measurement allows obtaining various landmarks: diameters of the aortic neck, aortic aneurysm, and both iliac arteries, aortic neck length, tortuosity of both iliac arteries, and angulation between the aortic neck and the aneurysm.

The evaluation of the semantic segmentation with AL showed that the average DSC and HD95 of the four classes became better or remained consistent as the stages progressed. In particular, the aorta consistently demonstrated superior performance compared to other classes across all stages. Saturation of the dataset was observed at Stage 1, with a reduction in segmentation time to 9.51 ± 1.02 min, indicating that the efficacy is closer to AI-assisted labeling techniques rather than active learning. However, for the thrombus, challenges occurred owing to the lack of contrast enhancement, making it difficult to discern surrounding structures. In addition, the vessels with thin walls posed a challenge owing to the low-resolution of the CT images. Notably, even in stage 5, on utilizing the entire dataset, a consistent performance improvement trend was observed. For calcification, which is characterized by small and randomly distributed patterns on the aortic wall, a relatively low performance was achieved. However, its relatively bright appearance led to saturation in stage 4. Despite the inferior performance compared to the aorta, they were observed that as the stages progressed, better performance was confirmed, indicating the efficacy of active learning.

**Figure 7:**
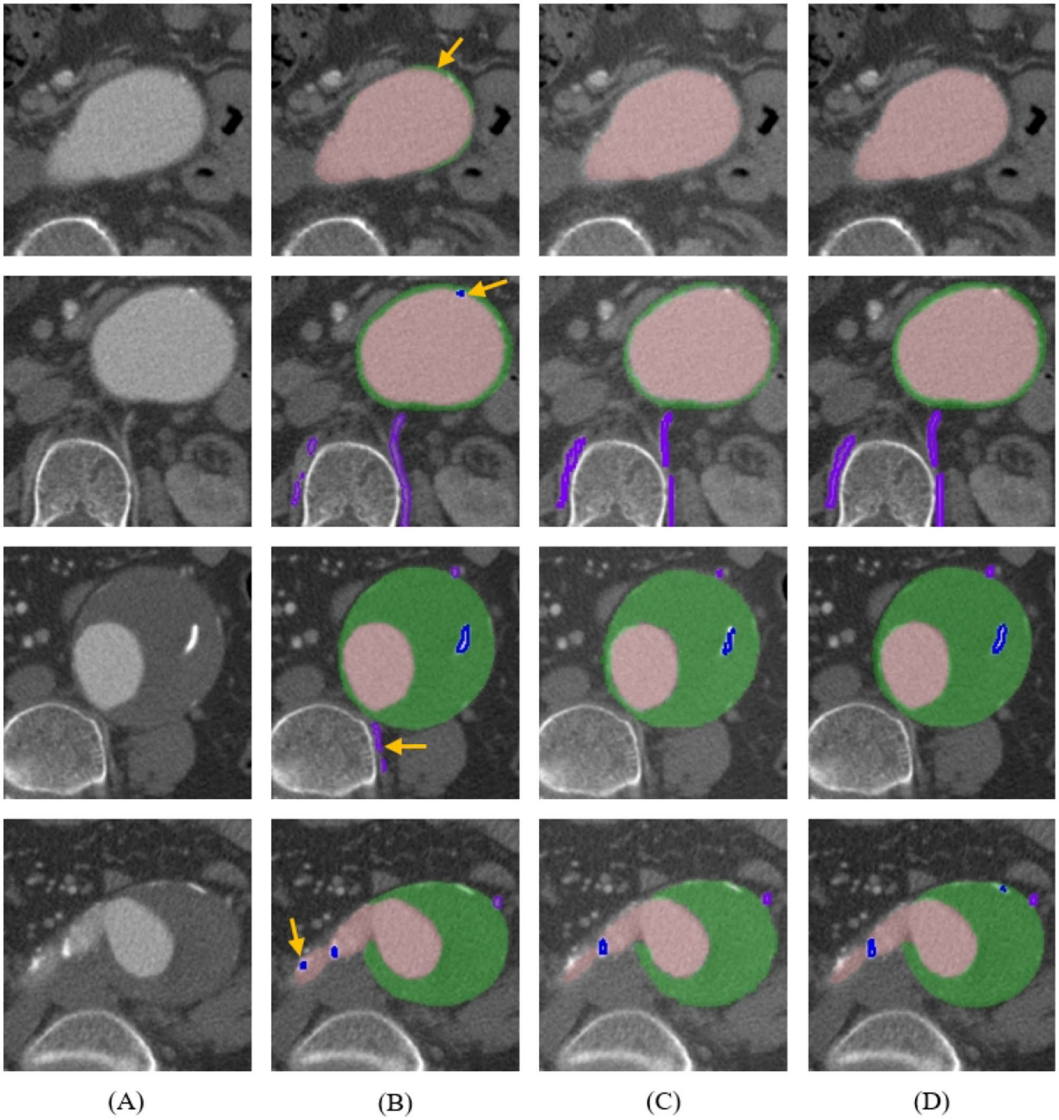
The challenge of labeling and false positive between ground truth and predictions. (**A**) Original CT images, (**B**) predictions of 3D U-Net, (**C**) predictions of SwinUNETR, and (**D**) ground truths. (Yellow arrow, false positive; Pink, aorta; Green, thrombus; Blue, calcification; purple; vessels).

We compared the predictions of the top-performing 3D U-Net, SwinUNETR, and the ground truths. False positives were identified in the challenging areas of the thrombus, calcification, and vessels where even manual labeling could be difficult (Fig. 7). In addition, AL-corrected segmentation significantly reduced the segmentation time by 13.74 ± 2.16 min compared to manual segmentation.

We developed two algorithms for automated measurement. The differences between manual and automated measurement can be attributed to their methodologies. The shortcomings of the traditional image-based manual measurement include challenges of accurately detecting the maximum diameter across multiple slices and three views, inadequate reproducibility, prolonged measurement time, and subjective determination of individuals. Our automated approach provides several benefits. First, it significantly decreases repetitive and labor-intensive manual tasks. Second, it reduces time-consuming processes, leading to increased efficiency. Third, it ensures consistency of both automated workflows and researchers. Finally, it can be applied in various medical applications.

There are several limitations in this study. First, the dataset was from only one institution; therefore, validation in multiple institutions is needed in future studies. Second, we found the best networks for each class using various networks in AL. However, to confirm the improved performance, experiments on optimized parameters such as augmentation and development of advanced networks are required. Finally, to achieve full automation, manual intervention needs to be minimized, and advanced algorithm development should be a focus of future research.

## Conclusions

We developed a semantic segmentation method using various networks with AL and automated measurement using API of CAD. The 3D U-Net demonstrated superior performance compared to the other networks. AL identified saturation stages for each class, and its time efficiency was verified. In addition, our automated measurement approach could be highly efficient in minimizing labor-intensive, time-consuming, and repetitive manual tasks.

### Supplementary Information

Supplementary Information.

Supplementary Video 1.

## Data Availability

The dataset could be available on request from the corresponding authors with allowance of our IRB.
