# Supplementary material for: Computed tomography-based automated measurement of abdominal aortic aneurysm using semantic segmentation with active learning
Source: Sci Rep. 2024 Apr 18;14:8924. doi: 10.1038/s41598-024-59735-8 (PMC11026521; doi:10.1038/s41598-024-59735-8)
Supplement: Supplementary file 1 — Supplementary Information. [file 41598_2024_59735_MOESM1_ESM.docx]

Supplementary Table 1. DSC for abdominal aortic aneurysm with UNETR and SwinUNETR and 2D U-Net, 3D U-Net, 2D–3D U-Net ensemble, and cascade 3D U-Net.

| Metric | Networks | Classes | Stage 1 | Stage 2 | Stage 3 | Stage 4 | Stage 5 |
| --- | --- | --- | --- | --- | --- | --- | --- |
| DSC | UNETR | Aorta | 0.890 ± 0.052 | 0.896 ± 0.042 | 0.906 ± 0.039 | 0.910 ± 0.037 | 0.913 ± 0.035 |
|  |  | Thrombus | 0.620 ± 0.226 | 0.665 ± 0.235 | 0.675 ± 0.234 | 0.697 ± 0.229 | 0.717 ± 0.195 |
|  |  | Calcification | 0.584 ± 0.179 | 0.645 ± 0.147 | 0.633 ± 0.182 | 0.664 ± 0.144 | 0.650 ± 0.153 |
|  |  | Vessels | 0.198 ± 0.150 | 0.312 ± 0.145 | 0.320 ± 0.149 | 0.325 ± 0.143 | 0.343 ± 0.140 |
|  | SwinUNETR | Aorta | 0.895 ± 0.050 | 0.904 ± 0.044 | 0.906 ± 0.043 | 0.916 ± 0.039 | 0.920 ± 0.036 |
|  |  | Thrombus | 0.660 ± 0.228 | 0.647 ± 0.234 | 0.678 ± 0.235 | 0.710 ± 0.249 | 0.736 ± 0.199 |
|  |  | Calcification | 0.611 ± 0.114 | 0.612 ± 0.180 | 0.622 ± 0.168 | 0.660 ± 0.157 | 0.669 ± 0.143 |
|  |  | Vessels | 0.240 ± 0.157 | 0.314 ± 0.159 | 0.358 ± 0.140 | 0.368 ± 0.147 | 0.364 ± 0.149 |
|  | 2D U-Net | Aorta | 0.899 ± 0.035 | 0.902 ± 0.038 | 0.911 ± 0.036 | 0.919 ± 0.027 | 0.922 ± 0.027 |
|  |  | Thrombus | 0.710 ± 0.206 | 0.699 ± 0.260 | 0.744 ± 0.200 | 0.752 ± 0.227 | 0.750 ± 0.223 |
|  |  | Calcification | 0.593 ± 0.214 | 0.605 ± 0.138 | 0.670 ± 0.230 | 0.693 ± 0.230 | 0.687 ± 0.230 |
|  |  | Vessels | 0.319 ± 0.142 | 0.294 ± 0.143 | 0.394 ± 0.185 | 0.395 ± 0.196 | 0.430 ± 0.186 |
|  | 3D U-Net | Aorta | 0.914 ± 0.035 | 0.913 ± 0.037 | 0.909 ± 0.039 | 0.918 ± 0.038 | 0.926 ± 0.027 |
|  |  | Thrombus | 0.733 ± 0.229 | 0.737 ± 0.228 | 0.746 ± 0.228 | 0.758 ± 0.230 | 0.779 ± 0.191 |
|  |  | Calcification | 0.674 ± 0.218 | 0.602 ± 0.161 | 0.665 ± 0.237 | 0.703 ± 0.199 | 0.702 ± 0.210 |
|  |  | Vessels | 0.310 ± 0.147 | 0.374 ± 0.168 | 0.437 ± 0.206 | 0.441 ± 0.191 | 0.481 ± 0.155 |
|  | 2D - 3D U-Net  ensemble | Aorta | 0.911 ± 0.032 | 0.910 ± 0.039 | 0.913 ± 0.035 | 0.922 ± 0.029 | 0.928 ± 0.026 |
|  |  | Thrombus | 0.732 ± 0.202 | 0.730 ± 0.239 | 0.762 ± 0.193 | 0.764 ± 0.226 | 0.769 ± 0.215 |
|  |  | Calcification | 0.643 ± 0.222 | 0.636 ± 0.133 | 0.690 ± 0.233 | 0.708 ± 0.224 | 0.702 ± 0.226 |
|  |  | Vessels | 0.320 ± 0.151 | 0.345 ± 0.161 | 0.411 ± 0.203 | 0.425 ± 0.195 | 0.453 ± 0.185 |
|  | Cascade  3D U-Net | Aorta | 0.914 ± 0.034 | 0.916 ± 0.035 | 0.916 ± 0.035 | 0.919 ± 0.033 | 0.926 ± 0.028 |
|  |  | Thrombus | 0.746 ± 0.189 | 0.748 ± 0.227 | 0.775 ± 0.178 | 0.756 ± 0.230 | 0.782 ± 0.170 |
|  |  | Calcification | 0.600 ± 0.228 | 0.680 ± 0.219 | 0.674 ± 0.226 | 0.710 ± 0.216 | 0.652 ± 0.244 |
|  |  | Vessels | 0.354 ± 0.142 | 0.420 ± 0.165 | 0.444 ± 0.156 | 0.469 ± 0.189 | 0.473 ± 0.171 |

Note: DSC, Dice similarity coefficient; UNETR, UNEt TRansformers; SwinUNETR, shifted-windows UNEt Transformers.

Supplementary Table 2. HD95 for abdominal aortic aneurysm with UNETR and SwinUNETR and 2D U-Net, 3D U-Net, 2D–3D U-Net ensemble, and cascade 3D U-Net.

| Metric | Networks | Classes | Stage 1 | Stage 2 | Stage 3 | Stage 4 | Stage 5 |
| --- | --- | --- | --- | --- | --- | --- | --- |
| HD95 | UNETR | Aorta | 6.661 ± 7.702 | 4.067 ± 5.607 | 3.950 ± 5.293 | 3.475 ± 4.999 | 3.765 ± 5.968 |
|  |  | Thrombus | 12.417 ± 17.777 | 12.239 ± 17.788 | 12.664 ± 18.275 | 10.302 ± 9.276 | 7.325 ± 6.160 |
|  |  | Calcification | 29.770 ± 12.506 | 21.613 ± 8.016 | 19.593 ± 7.682 | 20.114 ± 8.016 | 20.129 ± 7.285 |
|  |  | Vessels | 14.880 ± 12.678 | 11.451 ± 14.098 | 12.915 ± 15.654 | 12.902 ± 14.902 | 11.565 ± 12.541 |
|  | SwinUNETR | Aorta | 5.219 ± 8.608 | 3.913 ± 6.840 | 4.352 ± 5.726 | 2.832 ± 5.060 | 2.411 ± 3.836 |
|  |  | Thrombus | 10.388 ± 11.187 | 12.301 ± 11.321 | 15.028 ± 16.390 | 11.509 ± 13.286 | 8.958 ± 9.428 |
|  |  | Calcification | 22.903 ± 10.729 | 25.234 ± 12.621 | 17.492 ± 5.334 | 17.734 ± 6.732 | 17.086 ± 8.115 |
|  |  | Vessels | 13.531 ± 13.980 | 12.244 ± 12.494 | 16.265 ± 25.152 | 12.069 ± 12.538 | 12.465 ± 14.621 |
|  | 2D U-Net | Aorta | 5.319 ± 7.968 | 3.110 ± 2.163 | 2.744 ± 1.514 | 2.696 ± 1.947 | 3.145 ± 3.696 |
|  |  | Thrombus | 14.198 ± 12.952 | 15.475 ± 20.299 | 15.894 ± 26.019 | 14.790 ± 21.561 | 14.268 ± 18.339 |
|  |  | Calcification | 22.301 ± 18.637 | 15.820 ± 13.956 | 14.207 ± 18.799 | 14.329 ± 18.923 | 14.198 ± 18.830 |
|  |  | Vessels | 38.897 ± 21.549 | 24.976 ± 11.258 | 36.475 ± 21.386 | 38.530 ± 23.859 | 32.299 ± 19.254 |
|  | 3D U-Net | Aorta | 2.773 ± 2.277 | 2.002 ± 1.281 | 3.716 ± 5.750 | 3.299 ± 5.722 | 2.887 ± 2.761 |
|  |  | Thrombus | 17.282 ± 21.893 | 12.743 ± 16.377 | 18.851 ± 22.958 | 17.590 ± 26.293 | 12.313 ± 17.021 |
|  |  | Calcification | 13.244 ± 17.024 | 13.143 ± 13.375 | 23.783 ± 33.357 | 12.462 ± 15.024 | 12.391 ± 16.620 |
|  |  | Vessels | 46.720 ± 24.230 | 19.950 ± 11.205 | 24.647 ± 16.442 | 25.698 ± 16.723 | 23.384 ± 16.196 |
|  | 2D - 3D U-Net  ensemble | Aorta | 3.098 ± 2.547 | 2.223 ± 1.540 | 2.474 ± 1.407 | 2.177 ± 1.257 | 2.297 ± 1.423 |
|  |  | Thrombus | 14.198 ± 13.545 | 14.192 ± 20.182 | 15.309 ± 21.844 | 16.037 ± 23.098 | 14.054 ± 18.409 |
|  |  | Calcification | 15.637 ± 18.418 | 13.203 ± 13.848 | 14.551 ± 18.510 | 13.595 ± 19.086 | 13.251 ± 18.662 |
|  |  | Vessels | 45.648 ± 25.265 | 28.287 ± 20.943 | 35.348 ± 25.069 | 36.101 ± 30.467 | 33.239 ± 26.079 |
|  | Cascade  3D U-Net | Aorta | 2.549 ± 1.452 | 2.408 ± 1.352 | 2.601 ± 1.723 | 2.487 ± 1.707 | 2.686 ± 2.468 |
|  |  | Thrombus | 13.552 ± 14.101 | 18.755 ± 23.432 | 13.813 ± 18.054 | 17.496 ± 25.001 | 11.616 ± 13.021 |
|  |  | Calcification | 32.741 ± 42.828 | 15.990 ± 18.833 | 16.948 ± 25.194 | 12.009 ± 17.714 | 59.690 ± 67.151 |
|  |  | Vessels | 31.446 ± 17.306 | 35.840 ± 20.643 | 23.815 ± 14.934 | 17.645 ± 12.166 | 20.616 ± 11.933 |

Note: HD95, HD95, 95% Hausdorff distance; UNETR, UNEt TRansformers; SwinUNETR, shifted-windows UNEt Transformers

Supplementary Table 3. The segmentation time for manual and correction using 3D U-Net of nnU-Net with randomly selected 10 patients.

| Time  (min) | Manual segmentation | | | |  | AL-corrected segmentation (3D U-Net) | | | |  | p |
| --- | --- | --- | --- | --- | --- | --- | --- | --- | --- | --- | --- |
|  | Aorta | Thrombus | Calcification | Vessels |  | Aorta | Thrombus | Calcification | Vessels |  |  |
| 1 | 23.43 | 7.20 | 6.93 | 5.38 |  | 12.58 | 4.43 | 3.75 | 4.73 |  | - |
| 2 | 20.95 | 7.55 | 5.70 | 6.63 |  | 12.10 | 3.32 | 3.82 | 5.38 |  |  |
| 3 | 22.43 | 7.78 | 6.52 | 6.20 |  | 13.33 | 5.12 | 4.40 | 4.83 |  |  |
| 4 | 21.30 | 6.68 | 5.17 | 5.72 |  | 11.57 | 4.37 | 4.72 | 4.80 |  |  |
| 5 | 22.72 | 5.87 | 5.47 | 6.35 |  | 13.12 | 4.50 | 5.78 | 4.53 |  |  |
| 6 | 23.85 | 6.42 | 4.52 | 5.15 |  | 12.33 | 3.68 | 4.77 | 4.53 |  |  |
| 7 | 22.25 | 6.55 | 5.27 | 5.03 |  | 13.05 | 4.32 | 4.73 | 5.52 |  |  |
| 8 | 21.45 | 5.47 | 6.00 | 5.48 |  | 13.63 | 4.57 | 3.98 | 5.82 |  |  |
| 9 | 21.23 | 5.32 | 5.53 | 6.42 |  | 11.42 | 4.87 | 5.08 | 4.10 |  |  |
| 10 | 20.85 | 5.40 | 5.57 | 7.33 |  | 12.22 | 4.20 | 4.95 | 4.73 |  |  |
| Mean ± SD | 22.05±1.06 | 6.42±0.90 | 5.67±0.69 | 5.97±0.74 |  | 12.53 ± 0.74 | 4.34 ± 0.52 | 4.60 ± 0.62 | 4.90 ± 0.51 |  | <0.001 |

Note: Paired t-tests between manual and AL-corrected segmentation of 3D U-Net in segmentation time, AL; active learning, SD; standard deviation.

Supplementary Video S1. The automated measurement process
